# Cauterisation of the Neck of the Uterus

**Published:** 1842-07-16

**Authors:** 


*Cauterisation of the Neck of the Uterus.*—During the last three months of the past year M. Lisfranc has made a great number of experiments at the hospital of la Pitié, for the purpose of determining what is the best caustic that can be employed in cases where it may be necessary to cauterise the neck of the uterus. Simple ulcerations of this organ require the use of caustic, and it is now well ascertained that they are more or less rapidly healed by this means. Practitioners are equally in the habit of employing the nitrate of silver and the deuto-nitrate of mercury, but the circumstances which should guide us in the choice of either remedy have not been pointed out. From the numerous experiments instituted by M. Lisfranc, it would appear that hæmorrhage very rarely ensues after the use of the deuto-nitrate, while lunar caustic frequently occasions more or less abundant loss of blood. Hence it follows that, whenever ulceration of the neck of the uterus is accompanied, as it often is, by congestion or sub-inflammation of the organ, we must not employ the nitrate of silver, which has a tendency to increase the congestive state of the uterus.

During the last three months of the year 1841, 72 cauterisations were performed on eleven women affected with ulceration of the neck of the uterus; in 44 of these 72 operations, the nitrate of silver was used, and in 31 its employment was followed by a discharge of blood; on the other hand, the deuto-nitrate of mercury was used in 28 cases, and in three only occasioned a slight discharge.—*Ibid., from Bul. de. Therap.*

